# Prévalence et Déterminants de L'infection à Chlamydia Trachomatis chez les Femmes Consultant en Centres de Dépistage à la Réunion: Une Étude Transversale

**DOI:** 10.48327/mtsibulletin.n1.2021.69

**Published:** 2021-03-15

**Authors:** C. Duval, N. Anthony, E. Thore-Dupont, J. Jaubert, G. Camuset, P. Von Theobald, J.-M. Franco, P. Poubeau, L. Bruneau, A. Bertolotti

**Affiliations:** 1 CHU Réunion, Service des maladies infectieuses - dermatologie, Saint Pierre, La Réunion, France; 2 Inserm CIC1410, CHU Réunion, Saint Pierre, La Réunion, France; 3 CHU Réunion, Centre gratuit d'information de dépistage et diagnostic des IST, Saint Paul, La Réunion, France; 4 CHU Réunion, Laboratoire de microbiologie, Saint Pierre, La Réunion, France; 5 CHU Réunion, Service de gynécologie - obstétrique, Saint Denis, La Réunion, France; 6 Département de médecine générale universitaire, La Réunion, France

**Keywords:** *Chlamydia trachomatis*, Infections sexuellement transmissibles, Prévalence, Dépistage, Saint-Paul, Saint-Pierre, La Réunion, Océan Indien, *Chlamydia trachomatis*, Sexually transmitted infection, Prevalence, Screening, Saint-Paul, Saint-Pierre, La Réunion, Indian Ocean

## Abstract

**Introduction:**

L'infection à *Chlamydia trachomatis* (CT) est l'infection sexuellement transmissible (IST) bactérienne la plus répandue dans le monde. Souvent asymptomatique, elle peut mener à des complications significatives chez les femmes. L'objectif de cette étude était de déterminer la prévalence du CT en centre de dépistage à la Réunion et d'explorer les déterminants de cette infection.

**Méthode:**

Enquête transversale comprenant un questionnaire anonyme et une PCR par auto-écouvillon génital mené chez les femmes consultant dans deux centres de dépistage à la Réunion au cours d'une année.

**Résultats:**

Parmi les 620 femmes testées, la prévalence du CT était de 6,6% (IC 95% [4, 7, 8, 6]). La prévalence maximale par classe d'âge se situait entre 12 et 17 ans avec une prévalence de 14,3% contre 7,5% chez les 18-24 ans et 3,9% chez les 25-67 ans (p = 0,003). Les facteurs de risques étaient le jeune âge (p = 0,02), un premier rapport sexuel entre 11 et 14 ans (p = 0,01), une absence d'antécédent de dépistage (p = 0,02) et les motifs de consultation « infidélité du partenaire » (p = 0,01) et « partenaire infecté » (p = 0,02).

**Conclusion:**

La prévalence du CT chez les mineures réunionnaises est élevée. Un dépistage plus systématique et un renforcement de la sensibilisation aux IST chez elles semblent primordiaux.

## Abstract (en)

**Introduction:**

*Chlamydia trachomatis* (CT) infection is the commonest bacterial sexually transmitted infection (STI) in the world. Often asymptomatic, it can lead to significant complications in women. In France, since 2003, systematic screening for CT in STI center has been recommended for women aged less than 25 year. The main objective of this study was to determine CT prevalence in patients attending STI centers in Reunion Island. The second objective was to explore the determinants of this infection.

**Method:**

A cross-sectional survey using an anonymous questionnaire was conducted among women attending STI center in two hospitals in western and southern Reunion Island during one year. All women who had performed a CT PCR, based on vaginal self-swabs, were included.

**Results:**

Among the 620 patients tested, the prevalence of infection was 6.6% (95% CI [4.7-8.6]). By age group, the highest prevalence was between 12 and 17 years with 14.3% positive tests compared to 7.5% and 3.9% respectively in 18-24 and 25-67 year age group (p = 0.003). The risk factors for CT were a young age (p = 0.02), a first sexual intercourse between 11 and 14 years old (p = 0.01), lack of previous STI screening history (p = 0.02), and the following motives for seeking screening: “partner unfaithfulness” (p = 0.01) and “infected partner” (p = 0.02).

**Conclusion:**

This study highlights the high prevalence of CT among Reunionese minors. A more systematic screening and a reinforcement of STI awareness among young people in Reunion Island seem to be essential.

## Introduction

L'infection à *Chlamydia trachomatis* (CT) est l'infection bactérienne sexuellement transmissible (IST) la plus répandue dans le monde avec une incidence mondiale annuelle estimée à 105,7 millions [20]. Le plus souvent asymptomatique, cette infection peut se compliquer de douleurs chroniques, de grossesse extra-utérine et d'infertilité tubaire [17, 25]. Les données françaises du réseau Rénachla, montrent que le nombre d'infections à CT déclarées a augmenté de 15% entre 2014 et 2015 et diminué de 7% entre 2015 et 2016 [19]. Les techniques de biologie moléculaire avec amplification génique et les prélèvements non invasifs permettent de faciliter le dépistage et la prise en charge de cette infection [4, 10]. En 2003, l'Agence nationale d'accréditation et d'évaluation en Santé (remplacée en 2004 par la Haute Autorité de Santé) préconisait un dépistage préférentiel des femmes de moins de 25 ans (si l'objectif premier était la diminution des taux de complications) ou un dépistage simultané des hommes de moins de 30 ans et des femmes de moins de 25 ans (si l'objectif était la diminution du portage de CT), en précisant que le dépistage pouvait être élargi aux sujets ayant plus d'un partenaire sexuel dans l'année, quel que soit l'âge [9]. La stratégie de dépistage a été modifiée en 2018 par la Haute Autorité de Santé avec les recommandations suivantes: un dépistage opportuniste systématique des femmes sexuellement actives de 15 à 25 ans (inclus), y compris les femmes enceintes et un dépistage opportuniste ciblé chez les hommes sexuellement actifs présentant des facteurs de risques, les femmes de plus de 25 ans présentant des facteurs de risque et les femmes consultant pour une IVG [11].

À la Réunion, la surveillance du gonocoque et de la syphilis est effectuée par Santé publique France – Antenne océan Indien, en lien avec l'Agence régionale de santé de l'océan Indien avec le réseau « RésIST-Réunion ». Ce réseau, n'étant que peu étendu à la médecine de ville, sous-estime le poids de ces IST à la Réunion [3].

Concernant les réseaux de laboratoires dédiés aux infections à CT (Rénachla) et gonocoque (Rénago), ils n'ont pas, pour le moment, été mis en place.

Dans l'attente de pouvoir réaliser une étude en population générale, l'objectif principal de cette étude était de déterminer la prévalence de CT chez les patientes se présentant dans les centres de dépistage des IST Ouest et Sud de la Réunion au cours d'une période d'un an. L'objectif secondaire de cette étude était de déterminer les facteurs socio-démographiques et cliniques associés à l'infection à CT.

## Méthodologie

Il s'agit d'une étude épidémiologique, descriptive, bicentrique, transversale, menée dans les centres de dépistage du Centre hospitalier Gabriel Martin de Saint-Paul et du Centre hospitalier universitaire Sud de Saint-Pierre de la Réunion de septembre 2014 à août 2015. Les prélèvements éligibles étaient les auto-prélèvements vaginaux réalisés par les patientes ayant consulté dans ces centres durant cette période et pour lesquelles une recherche de CT avait été demandée. Etaient exclus les prélèvements dont le résultat CT était indisponible, les prélèvements extra-génitaux, les prélèvements itératifs pour une même patiente et les patientes ne vivant pas à la Réunion. La recherche de CT se faisait sur des auto-écouvillons vaginaux. Chaque centre complétait, le jour de la venue de la patiente, un questionnaire anonyme après consentement de la patiente (ou du majeur responsable), permettant de recueillir des données socio-démographiques et cliniques. Les variables qualitatives ont été décrites en termes de fréquence et pourcentage. Les variables quantitatives ont été exprimées en moyenne et écart-type. Les comparaisons de moyennes étaient réalisées à l'aide d'un test t de Student ou de Mann-Whitney si les conditions de validité n'étaient pas remplies. Les variables qualitatives étaient comparées à l'aide du test du Chi2 ou du test exact de Fisher selon les conditions d'applications. Le seuil de significativité retenu pour l'ensemble des tests a été de 0,05. Les analyses étaient réalisées avec le logiciel SAS 9.4^®^. Aucune imputation des données manquantes n'a été effectuée. Cette étude était réalisée selon la méthodologie de référence MR-004 de la Commission nationale de l'informatique et des libertés. Le consentement éclairé des participantes a été recueilli et les données ont été traitées de manière anonyme. Cette étude a été enregistrée dans le registre de l'Institut national des données de santé sous le numéro MR 0314090620.

## Résultats

**Fig. 1:**
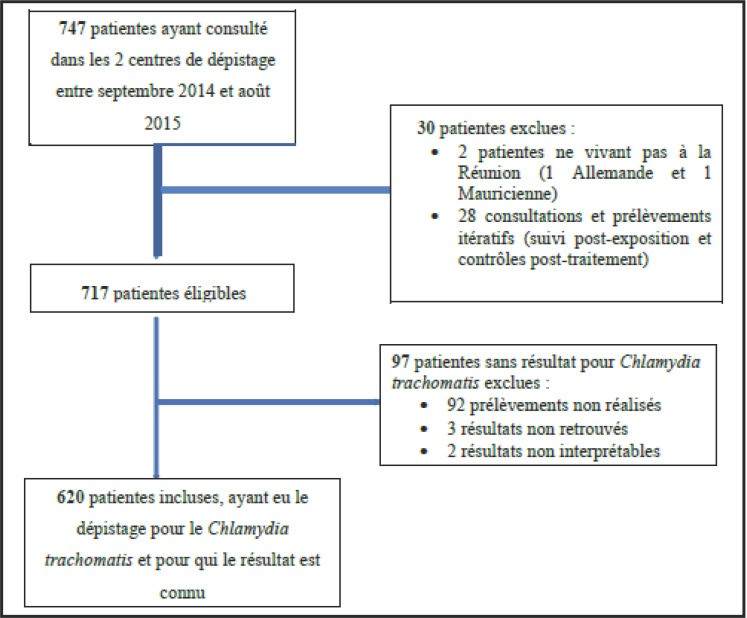
Diagramme de flux Flow chart

Entre septembre 2014 et août 2015, 747 patientes ont consulté dans ces deux centres. Deux personnes ne vivant pas à la Réunion ont été exclues. Vingt-huit patientes avaient réalisé des prélèvements itératifs, soit dans le cadre d'un suivi post-exposition, soit dans le cadre d'un contrôle post-traitement. Les contrôles itératifs ayant tous été négatifs pour les dépistages IST, seule la première consultation et le premier prélèvement ont été pris en compte. Quatre-vingt-dix-sept patientes ont été exclues de l'étude pour cause de non-réalisation du prélèvement ou de résultat de CT non retrouvé ou ininterprétable. Au total, chez les 620 patientes inclues et dépistées pour l'infection par CT, 41 patientes ont eu un prélèvement positif (Figure 1). La plus jeune patiente de l'échantillon infectée à CT avait 14 ans et la plus âgée 52 ans.

La prévalence était de 6,6% (IC 95% [4, 7, 8, 6]) (Tableau 1). Il n'y avait pas de différence significative entre les lieux de dépistage (p = 0,72).

**Tableau I:** Déterminants de l'infection à *Chlamydia trachomatis* chez les femmes consultant en centre de dépistage Determinants of Chlamydia trachomatis infection in women attending screening centres

		Effectif	CT+ N = 41 (6,6%)	CT- N = 579 (93,4%)	p
**Âge en années moyenne (écart-type)**		547	23 (8,9)	26,8 (9,6)	0,02
**Classes d'âge**		572			0,003
	12-17 ans		13 (33,3%)	78 (14,6%)	
	18-24 ans		15 (38,5%)	184 (34,5%)	
	25-67 ans		11 (28,2%)	271 (50.8%)	
**Couverture sociale**		178			1
	Sécurité sociale		7 (70%)	113 (67,3%)	
	CMU		3 (30%)	55 (32,7%)	
**Lieu du dépistage**		620			0,72
	Saint Paul		18 (43,9%)	271 (46,8%)	
	Saint Pierre		23 (56,1%)	308 (53,2%)	
**Antécédent d'IST**		479	4 (12,9%)	79 (17,6%)	0,5
**Présence de symptômes**		503	5 (15,6%)	79 (16,8%)	0,87
**Vaccination hépatite B**		579	28 (73,7%)	370 (68,4%)	0,5
**Antécédent de FCS ou d'infertilité**		269	1 (5%)	29 (11,6%)	0,71
**Partenaire fixe**		540	25 (64,1%)	302 (60,3%)	0,64
**Port du préservatif**		450			0,81
	Jamais		10 (30,3%)	98 (23,5%)	
	Occasionnellement		6 (18,2%)	74 (17,8%)	
	Souvent		10 (30,3%)	134 (32,1%)	
	Toujours		7 (21,2%)	111 (26,6%)	
**Âge du premier rapport sexuel**		246			0,01
	11-14 ans		5 (27,8%)	13 (5,7%)	
	15-17 ans		7 (38,9%)	134 (58,8%)	
	18-25 ans		6 (33,3%)	81 (35,5%)	
**ATCD de dépistage IST**		537	18 (46,1%)	321 (64,5%)	0,02
**Date du dernier dépistage**		312			0,63
	Moins d'un an		5 (27,8%)	95 (32,3%)	
	Entre un et deux ans		3 (16,7%)	69 (23,5%)	
	Plus de 2 ans		10 (55,5%)	130 (44, %)	
**Dépistage réalisé dans le cadre d'un rapport à risque**		505 505	13 (36,1%)	197 (42%)	0.49
**Dépistage réalisé pour un bilan sans prise de risque**		505	16 (44,4%)	158 (33,7%)	0,19
**Dépistage réalisé pour l'arrêt du préservatif**		505	5 (13,9%)	85 (18,1%)	0,52
**Dépistage réalisé en cas de doute de la fidélité du partenaire**		254	4 (23,5%)	11 (4,6%)	0,01
**Dépistage réalisé car partenaire porteur d'une IST**		505	4 (11,1%)	11 (2,3%)	0,02
**Dépistage réalisé pour signes cliniques**		505	2 (5,6%)	30 (6,4%)	1
**Dépistage réalisé pour d'autres motifs**		505	2 (5,6%)	49 (10,5%)	0,56
**Contraception**		279	11 (61,1%)	129 (49,4%)	0,34
**Test réalisé à 2**		230	6 (42,9%)	86 (60,2%)	0,82
**Suivi de la patiente**		286	13 (72,2%)	228 (85,1%)	0,17

Les patientes infectées par CT étaient plus jeunes que les patientes non infectées: respectivement 23 ans versus 27 années (p = 0,02). Parmi les patientes positives, plus de 70% avaient moins de 25 ans. La classe d'âge comprenant la plus haute prévalence de l'infection était celle de 12-17 ans avec 14,3% d'infections contre 7,5% chez les 17-24 ans et 3,9% chez les 25-67 ans (p = 0,003).

Les femmes ayant eu leur premier rapport sexuel entre 11 et 14 ans avaient significativement plus d'infections à CT que celles ayant eu leur premier rapport entre 15 et 17 ans ou entre 18 et 25 ans (p = 0,01).

La proportion de femmes n'ayant jamais fait de dépistage des IST au cours de leur vie était significativement plus importante chez les femmes CT+ que chez les femmes CT- (p = 0,02).

Concernant les motifs de consultation (Tableau 1), les femmes CT+ consultaient significativement plus que les femmes CT- dans le cadre d'un doute sur la fidélité de leur partenaire (p = 0,01) ou dans le cadre d'une infection sexuellement transmissible chez le partenaire (p = 0,02).

## Discussion

La prévalence de CT retrouvée chez les patientes était de 6,6%, prévalence comparable aux données de France métropolitaine (entre 5,5 et 14,5%) [1, 15, 24]. La spécificité de la Réunion réside dans la prévalence de l'infection particulièrement élevée (14,3%) chez les adolescentes de 12 à 17 ans. Cela souligne les comportements sexuels à risque de ces jeunes patientes réunionnaises.

Cette prévalence se rapproche de la prévalence de 16,1% identifiée dans un échantillon d'adolescentes à haut risque en métropole [8]. Par ailleurs, l'enquête ETADAR en 2006/2007 confirmait déjà cette prise de risque chez les adolescents (garçons et filles) réunionnais [5]. Le nombre important de grossesses précoces désirées ou non et d'IVG chez les mineures à la Réunion par rapport à la métropole souligne également le manque de connaissance des patientes mineures en matière de sexualité et de contraception favorisant ces comportements à risque [6, 22]. La persistance d'idées fausses chez les jeunes concernant la transmission des IST rend primordiale leur éducation en matière de contraception et de sexualité [18, 21]. Cette forte prévalence retrouvée chez les 12-17 ans ainsi que la précocité du premier rapport sexuel comme facteur de risque justifieraient ainsi la réalisation d'études complémentaires en population générale et au sein des collèges et lycées, ainsi qu'une meilleure sensibilisation des adolescents à la transmission et aux risques des IST. L'éducation sexuelle est inscrite dans la loi depuis 2001 (article L312-16 du code de l'éducation). Au moins trois séances annuelles de cours d'éducation sexuelle aux écoles, collèges et lycées sont prévues par la loi. Cependant la circulaire reste peu appliquée d'après une enquête réalisée par le Haut conseil à l'égalité entre les femmes et les hommes (HCE) en 2016 [12]. Une étude sur l'impact médico-économique de l'infection à CT serait pertinente à la Réunion au vu de la prévalence importante et du fait des risques de séquelles néfastes sur la fertilité ultérieure des jeunes femmes. Une telle étude pourrait ainsi encourager la mise en place de nouvelles mesures d'information, de prévention et de dépistage ciblé.

Au vu des données de l'étude, la bonne application des recommandations HAS 2018 en termes de dépistage chez les jeunes personnes sexuellement actives (mineurs inclus) reste primordiale.

Hormis le jeune âge de la patiente et la précocité du premier rapport sexuel, d'autres facteurs étaient associés de façon significative à l'infection à CT: l'absence d'antécédent de dépistage et les motifs de consultation suivants: « doute sur la fidélité du partenaire » et « partenaire porteur d'une IST ». Le motif « partenaire porteur d'une IST » associé à l'infection à CT souligne l'importance des recommandations qui préconisent de traiter systématiquement le(s) partenaire(s) sexuel(s) d'une personne infectée [10].

Les patientes dans cette étude ont été incluses sur la base d'un prélèvement par auto-écouvillon vaginal. D'autres types de prélèvement tel que le premier jet d'urines ou le prélèvement endocervical par un médecin auraient pu être envisageables mais l'auto-prélèvement possède de nombreux avantages faisant de lui un examen de dépistage à favoriser. En effet, il est très simple à réaliser, ne nécessite pas d'examen gynécologique, a une très bonne acceptabilité en population et une performance diagnostique plus élevée que les deux autres examens [2, 7, 13, 14].

À la Réunion, les IST telles que la syphilis et la gonococcie sont en augmentation depuis 2014 avec respectivement 64 et 103 cas en 2018. Par contre, le VIH est assez bien contrôlé (94% des charges virales indétectables). La file active d'environ 1000 patients dans l'île et l'incidence de découverte restent stables depuis 10 ans (près de 50 nouveaux cas annuels). Par rapport à la France métropolitaine hors Ile-de-France, les nouveaux cas sont issus plus fréquemment de rapports homosexuels masculins (56,5% vs. 45,4%) et originaire de métropole (78,7% vs. 48,9%) [23]. La population concernée est donc bien distincte de celle décrite dans ce travail.

Le médecin généraliste, interlocuteur privilégié de santé chez les jeunes, est au coeur de cette lutte contre les IST en participant à l'éducation des patientes, au dépistage et au traitement précoce. Des études réalisées auprès de médecins généralistes confortent le constat d'insuffisance de dépistage de l'infection à CT en médecine générale avec, comme principaux obstacles au dépistage, l'absence de recommandations claires et la difficulté à aborder le sujet de la sexualité lors des consultations [16]. Informer les médecins généralistes sur les IST et notamment sur l'infection à CT parait primordial. La mise à disposition de kits d'auto-prélèvement pourrait également faciliter le dépistage de l'infection par les médecins généralistes car faciles à réaliser et bien acceptés par les patientes [6]. De façon plus générale, l'élaboration d'un système de surveillance régulier, tel que Rénachla, semble primordiale pour estimer le poids des infections à la Réunion.

Enfin, ces données provenant de centres de dépistage d'IST, l'extrapolation des résultats à l'ensemble de la population de femmes réunionnaises doit rester prudente. De plus, le taux de données manquantes de certaines variables, invite à la prudence concernant l'interprétation des résultats et notamment celle de la couverture sociale. En effet, le taux de précarité identifié dans cette étude, inférieur à celui des données régionales (32,4% vs. 44%), incite à explorer davantage le contexte socio-économique afin d'affiner le profil des patientes à risque lors de prochaines études. Il pourrait être en lien avec le manque d'éducation sexuelle précédemment cité, voire de difficulté de transport pour parvenir jusqu'au centre.

## Conclusion

Pour conclure, notre étude montre que la Réunion est également concernée par l'infection à CT chez les femmes. La situation à la Réunion se distingue par une prévalence particulièrement importante chez les mineures. Ceci souligne une fois de plus la prise de risque majeure de cette population. Un renforcement de la prévention en termes d'éducation sexuelle dans les collèges et les lycées semble particulièrement pertinent dans ce contexte.

## Conflits D'intérêts

Les auteurs ne déclarent aucun conflit d'intérêts.
